# Pharmacogenomic Testing to Guide Personalized Cancer Medicine Decisions in Private Oncology Practice: A Case Study

**DOI:** 10.3389/fonc.2020.00521

**Published:** 2020-04-28

**Authors:** George Astras, Christos I. Papagiannopoulos, Konstantinos A. Kyritsis, Constantina Markitani, Ioannis S. Vizirianakis

**Affiliations:** ^1^Department of Oncology, American Medical Center, Nicosia, Cyprus; ^2^Laboratory of Pharmacology, School of Pharmacy, Aristotle University of Thessaloniki, Thessaloniki, Greece

**Keywords:** personal cancer genome sequencing, precision medicine, oncology, pharmacogenomic testing, targeted therapeutics, next generation sequencing, routine clinical practice

## Abstract

Innovative tumor profiling methodologies are utilized to elucidate the pharmacogenomic landscape of tumor cells in order to support the molecularly guided delivery of therapeutics. Indeed, improved clinical outcomes are achieved in oncology practice by providing the physicians with expert-guided, standardized, and easily interpretable knowledge, translated from molecular profiling analysis to support clinical decision-making. However, there is still limited utilization of the technology especially in small private oncology practices. In this work, we analyzed how molecularly guided interventions in 17 consented cancer patients led to an overall improvement of disease response rates in a private oncology center. The precision medicine strategy was based on the OncoDEEP™ profiling solutions and focused on finding clinically actionable relationships between tumor biomarkers and drug responses. The obtained data support the notion that (a) following the pharmacogenomic-derived recommendations favorably impacted cancer therapy progression, and (b) the earlier profiling followed by the delivery of molecularly targeted therapy led to more durable and improved pharmacological response rates. Moreover, we report the example of a patient with metastatic gastric adenocarcinoma who, based on the molecular profiling data, received an off-label therapy that resulted in a complete response and a current cancer-free maintenance status. Overall, our data provide a paradigm on how molecular tumor profiling can improve decision-making in the routine private oncology practice.

## Introduction

Recent statistical studies concerning the incidence of malignancies propose that despite the clinical success obtained during the previous years, cancer is expected to be the leading cause barrier of increasing life expectancy in the 21st century (1, 2). This is, at least partially, attributed to the fact that cancer therapies are still applied on an experience-based manner and often without taking into consideration the special genomic and proteomic landscape of the tumor. Consequently, cancer therapy still fails to provide long-lasting outcomes, while many regimens are often toxic and costly for patients and healthcare systems. Personalized medicine in oncology strives to overcome those limitations by integrating genomic, transcriptomic, and proteomic analysis of tumor samples to decision-making in oncology. In a typical screening of such type, multiple genes and proteins implicated in tumor initiation, progression, and drug resistance are analyzed in tumor biopsies in order to identify potential alterations that may aid in therapy decision. The success of the technology has already been demonstrated for various combinations of altered biomarkers and therapeutic molecules, such as epidermal growth factor receptor (EGFR) expression and EGFR tyrosine kinase inhibitors/antibodies, or the expression of programmed death ligand 1 (PD-L1) with anti-PD1 and anti-PD-L1 therapies. This way, clinicians obtain a personalized therapeutic overview, which assists them to decide on the potential: (a) clinical benefit, (b) inefficacy, and (c) toxicity of a treatment. Moreover, they are informed on the potential utility of a drug with an off-label indication or about treatments that are under investigation in ongoing clinical trials.

Multiple studies have provided solid proof on the significance of molecular tumor profiling in precision cancer therapy. Initial efforts in the field have focused on tumor genomic profiling, based solely on Next-Generation Sequencing (NGS) technology, to show that such analysis may enable improved therapeutic outcomes (3, 4). Evidently, NGS analysis has resulted in the identification of a plethora of actionable variants that influence drug safety and efficacy in the clinical setting. Nowadays, ongoing studies and clinical trials include tissue genomic and transcriptomic analysis (5), while others also include immunohistochemistry, tumor mutational burden (TMB), and microsatellite instability (MSI) (6). Moreover, high-throughput methods in the fields of transcriptomics and proteomics are constantly being developed in parallel with sophisticated data analysis software tools. To date, all studies conclude that the molecular tumor profiling represents the best approach for obtaining information that would be useful to clinicians in routine oncology practice. The final goal would be to implement complex strategies of molecular profiling and guide the proper treatment approach for each patient independently in a cost-effective manner (7–10).

Despite the recent advances in profiling technology and the accumulating knowledge in the field of pharmacogenomics, personalized treatment care is still often missing especially in small oncology practices and centers. Toward this direction, the implementation of precision cancer medicine in routine private healthcare is presented in this work. The data analysis of 17 consented cancer patients are included, whom tumor biopsy samples were subjected to molecular profiling analysis to guide personalized therapy interventions. All patients provided a primary tumor sample for molecular analysis, after failing at least one standard of care treatment. By carefully monitoring each patient's response, we recorded patient cancer status before and after the initiation of the pharmacogenomics-based therapy. We report that the application of a personalized therapy, based on tumor profiling, favorably impacted response to therapy in 8 out of the 17 patients. We, also, describe the example of a patient with Stage IV metastatic gastric adenocarcinoma who demonstrated a complete response (CR) to an off-label molecularly targeted therapy and continued maintenance therapy with the proposed therapy while currently maintaining his CR status.

## Patients and Methods

A written informed consent was obtained from all the patients included in the present study (no minors exist within this group). In particular, the progression of 17 patients diagnosed with various types of cancer and consented to provide an excised primary tumor sample for molecular analysis, after failing at least one standard of care treatment, was monitored and reported. All types of molecular analysis on the patient tissue samples were conducted between March 2017 and November 2018 by applying the OncoDEEP™ (OncoDNA SA, Gosselies, Belgium) profiling solutions. The methodology applied in precision medicine strategy upon the patient sample handling, the DNA isolation, the NGS, the interpretation of the generated data, the identification of putative somatic mutations, as well as the analysis of immunohistochemistry (IHC) results, along with the clinical translation and implementation of the molecular data to the oncologist's evaluation report, has been previously described in detail (8). Importantly, all patients were informed about the OncoDEEP™ solutions and the suggested therapy by the medical oncologist and they consented before the tumor tissue sample was taken and sent for the molecular testing. The average age of the patients during their diagnosis was 52 years; 11 (~65%) of them were men and 6 (~35%) were women. The cancer types and the treatment schemes applied to patients based on routine clinical practice and after the OncoDEEP™ molecular profiling is presented in detail in Table 1.

**Table 1:** Patient information concerning personal information, malignancy type, and treatments received.

**Code**	**Diagnosed cancer type**	Age (range^*^) upon diagnosis	**Therapeutic scheme**	
			**Before the tumor profiling**	**After the tumor profiling**
CYP100	Colorectal cancer	60–70	(a) Xelox (b) FOLFOX	(c) FOLFIRI and Bevacizumab
CYP101	Ovarian cancer	40–50	(a) Carboplatin and Paclitaxel (b) Caelyx and Carboplatin (c) Carboplatin and Gemcitabine (d) Topotecan (e) Docetaxel	(f) Caeloyx
CYP102	Gastric cancer	40–50	(a) Xelox	(b) EOX (c) Pembrolizumab
CYP103	Carcinoma of unknown primary site	50–60	(a) Cisplatin and Capecitabine	(b) ECX (c) Nivolumab (d) Gemcitabine and Taxol
CYP104	Small cell lung cancer	70–80	(a) Cisplatin, Etoposide and Zometa (b) Paclitaxel and Zometa	(c) Topotecan weekly and Zometa
CYP105	Cervix adenocarcinoma	20–30	(a) Cisplatin and Etoposide (b) Radiotherapy/chemotherapy (c) Paclitaxel/Topotecan	(d) Carboplatin, Paclitaxel and Bevacizumab (e) CAV
CYP106	Cholangiocarcinoma	60–70		(a) Gemcitabine and Cisplatin (b) FOLFOX
CYP107	Pancreatic cancer	60–70	(a) FOLFIRINOX (b) Gemcitabine and Abraxane (c) Gemcitabine and Abraxane	
CYP108	Non-Small Cell Lung Cancer	60–70	(a) Cisplatin and Pemetrexed (b) Pemetrexed maintenance (c) Carboplatin/Paclitaxel/ Bevacizumab (d) Nivolumab (Opdivo)	
CYP109	Sarcoma	40–50	(a) Crizotinib (oral)	(b) Alectinib (oral) (c) Alectinib and Pembrolizumab
CYP110	Melanoma	30–40	(a) Ipilimumab (b) Pembrolizumab and Ipilimumab and Zometa x (c) Nivolumab and Ipilimumab and Zometa (d) Pembrolizumab and Ipilimumab and Zometa	(e) TIL Adoptive cell therapy (f) Pembrolizumab and Zometa (g) Carboplatin, Paclitaxel and Pembrolizumab
CYP111	Cholangiocarcinoma	60–70	(a) Gemcitabine and Cisplatin	
CYP112	Pancreatic cancer	40–50	(a) Gemcitabine and Abraxane (Nab-paclitaxel)	(b) Re-challenge Gemcitabine and Abraxane
CYP113	Thymoma and Thymic carcinoma	30–40	(a) Cyclophosphamide, Doxorubicin and Cisplatin (CAP) (b) Brachytherapy (c) CAP (d) CAP (e) Brachytherapy (f) Radiotherapy (g) Carboplatin and Etoposide	(h) Carboplatin, Paclitaxel and Bevacizumab
CYP114	Triple-negative breast cancer	50–60	(a) TDM1, Gemcitabine and Carboplatin	(b) TDM1, Paclitaxel and Carboplatin (c) Heceptin, Paclitaxel and Zometa (d) Capecitabin, Vinorelbine and Zometa
CYP115	Leiomyosarcoma	50–60	(a) Lartruvo and Doxorubicin (c) Brachytherpay	(b) Gemcitabine and Docetaxel
CYP116	Cholangiocarcinoma	60–70		(a) Gemcitabine and Cisplatin × 6 cycles (b) Gemcitabine maintenance × 2 cycles (c) CAP-OX (Capecitabine and Oxaliplatin

* *Age is shown as decade range for ensuring patient privacy*.

Moreover, we used Resist Criteria 1.1 for assessment and evaluation of therapy response (also called Response Evaluation Criteria In Solid Tumors). The types of response a patient can have are a complete response (CR), a partial response (PR), progressive disease (PD), and stable disease (SD). According to the guidelines, the scores are explained as follows: Complete Response (CR): Disappearance of all target lesions. Partial Response (PR): At least a 30% decrease in the sum of diameters of target lesions, taking as reference the baseline sum diameters. Progressive Disease (PD): At least a 20% increase in the sum of diameters of target lesions. Stable Disease (SD): Neither sufficient shrinkage to qualify for PR nor sufficient increase to qualify for PD. Moreover, we used the term Mixed Response (MR), which denotes that a tumor group located in a certain anatomical site responded to treatment but a different group in another anatomical site did not respond, reflecting the heterogeneity of tumors and differential response in different organs.

## Results

### Analysis of the Mutations Identified in the Patient Samples

Investigation of the genomic landscape of a tumor through NGS analysis allows the identification of actionable variants that promote tumor initiation and progression. Accumulating research over the past decade led to the clinical validation of multiple variants as pharmacogenomic biomarkers. With OncoDEEP™, each patient sample was screened for mutations in 65 genes that have evidently been related to cancer. At least one mutation was identified in 14 out of the 17 patients analyzed; 2 patients had no mutations (within the panel of the genes tested by NGS analysis) while the analysis failed for 1 patient due to bad sampling. In total, 25 unique mutations were identified in the group of patients, with Rat (RAS) sarcoma isoforms (6 mutations) and the tumor protein p53 (TP53, 4 mutations) being the most frequently mutated genes (Table 2; Supplementary Figure 1). Additionally, 21 out of the 25 (84%) mutations were classified as damaging, denoting that these variants had a known functional impact (activating or inhibiting) on the protein encoded, and this is supported by both research and clinical information. The rest of the variants (4 out of 25, 16%) were classified as potentially damaging, thus denoting that for those variants, there may be *in vitro* information but no clinical data supporting a role in altering protein function. As for the mutational burden of the tumor, most patients demonstrated a single or no mutation (11 out of 16), whereas 3 patients had between 2 and 3 mutations. Conversely, a patient with small-cell lung cancer demonstrated the highest number of mutations identified in a single tumor with five mutations presenting in key genes driving tumor progression (PIK3CA, JAK3, TP53, FGFR4, and JAK2). An overview of the mutated genes and the total number of patients bearing each mutation are shown in Table 2.

**Table 2:** Total number of mutations identified in the patients' cancer genome.

**Mutated gene**	**Number of patients with mutation**	**Mutation type** (red: damaging mutations; blue: potentially damaging mutations)	**Patient code**
RAS	6	c35G>A c.181C>A c.34G>C c.35G>T	CY100 CY102 CY103 CY106, CY107, CY112
TP53	4	c.818G>A c.202G>T c.586C>T c.504del	CY101 CY108 CY112 CY114
PIK3CA	3	c.263G>A c.1636C>A c.3140A>G	CY102 CY108 CY114
TPMT	2	c.719A>G c.460G>A	CY103 CY112
RB1	1	c.2148_2156del	CY104
GNAS	1	c.2531G>A	CY105
CDKN2A	1	c.210_211insC	CY106
JAK3	1	c.2164G>A	CY108
JAK2	1	c.1666T>G	CY108
FGFR4	1	c.2018G>A	CY108
SMO	1	Genomic amplification	CY110
AKT1	1	c.49G>A	CY114
SMAD4	1	c.346C>T	CY114
PMS2	1	c.1866G>A	CY116

The generated NGS data and the variants identified were used in order to advice on a potential therapy for the patients. For instance, mutations in the KRAS oncogene locus relate with resistance to an anti-epidermal growth factor receptor (anti-EGFR) therapy, thereby connecting such a treatment with poor clinical benefit and, thereby, the oncologist was discouraged from choosing it (11, 12). Similarly, a damaging thiopurine methyltransferase (TPMT) variant was used in order to exclude a cisplatin therapy in a patient with pancreatic cancer, as reduced metabolism of the drug due to the variant would lead to enhanced toxicity for that patient. Finally, the NGS analysis identified genomic amplification of the smoothened homolog (SMO) gene in a melanoma patient and thereby SMO inhibitors (sonidegib and vismodegib) were suggested as a treatment of choice for that cancer (13). The described examples underline the importance of investigating the genomic landscape of cancer before deciding on a proposed therapy.

### Molecular Analysis of Protein Pharmacogenomic Biomarkers

Similar to genetic biomarkers, the analysis of common biomarkers of proteinaceous nature is highly informative in personalized cancer therapy. Examples of such biomarkers include the elevated expression of Topoisomerase I and 4E-Binding protein (p4E-BP1), which relate to a beneficial response to Topoisomerase 1 inhibitors and PI3K/mTOR inhibitors, respectively (14, 15). On the contrary, multiple studies suggest that increased expression of the excision repair complementation group 1 (ERCC1) protein induces resistance to platinum-based chemotherapy (16–18). In total, the expression and the presence of multiple proteins and biomarkers that have evidently been connected to response to a particular therapy were analyzed (all biomarkers are shown in detail in Supplementary Figures 2, 3). Notably, at the protein level, p4E-BP1 and ERCC1 were found to be frequently upregulated (Supplementary Figure 2). These findings suggest that those patients may receive mammalian target of rapamycin (mTOR) inhibitors, but they should avoid a platinum-based chemotherapy due to ERCC1 overactivation, which would undermine the therapeutic outcome. Moreover, multiple patients demonstrated high expression of the cluster of differentiation 8 (CD8) protein, thus suggesting that a therapy with anti-CD8 (checkpoint) inhibitors would be a beneficial regimen. In total, the data generated from known protein biomarkers constituted a common strategy to choose a potential therapy in the group of 17 patients.

### Clinical Response of Patients Receiving Personalized Treatment Based on the Tumor Genome Molecular Analysis

**Figure 1:**
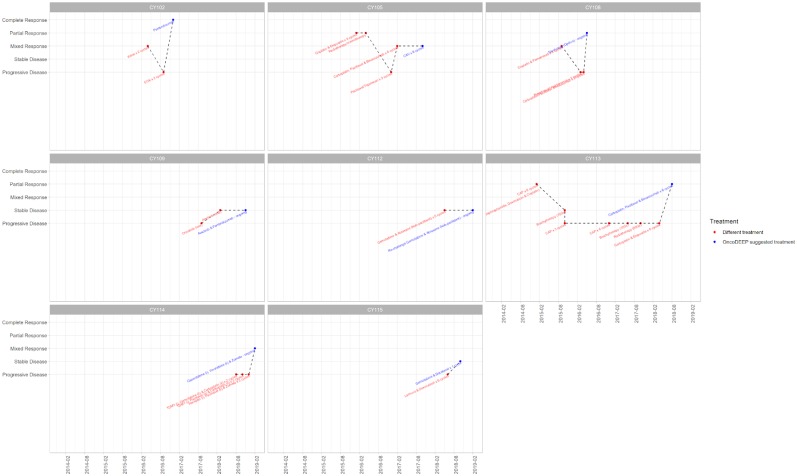
Treatment record for the eight patients that showed positive response to the suggested treatment. The *x*-axis displays a timeline (per 6 months), while the *y*-axis shows the response of the patient before applying the displayed treatment. Therapy regimens given before the personalized assessment are shown in red, whereas the personalized treatment is highlighted with blue. Plots were created using the ggplot2.

**Figure 2:**
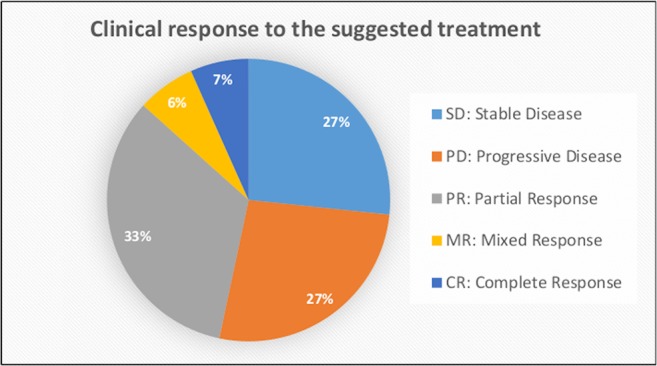
Data analysis showing the clinical response to the personalized treatment for all patients included in this study.

The outcome of the OncoDEEP™ analysis is a report that includes a list of therapies associated with (a) clinical benefit, (b) potential lack of benefit, or (c) toxicity. To evaluate the outcome of the suggested therapy for the group of 17 patients, we monitored how each patient responded to the applied therapy. For that, we used Resist Criteria 1.1 to classify the therapeutic outcome into the following: Complete Response (CR), Partial Response (PR), Mixed Response (MR), Progressive Disease (PD), and Stable Disease (SD); criteria are explained in detail under the Patients and methods section). Interestingly, we report that 8 out of the 17 patients (47%) showed an overall positive response to the molecularly guided therapy. The detailed response records for these eight patients are shown in Figure 1. Therapy regimens given before the personalized assessment are shown in red, whereas the personalized treatment is highlighted with blue. Moreover, we observe that the earlier a molecularly guided therapy is applied, the more beneficial the therapeutic outcome was. This is evident when comparing the treatment responses between the groups of CY114–CY102–CY115 to that of CY113–CY115. In particular, patient CY113 failed to respond to seven rounds of anti-cancer therapies, before receiving a pharmacogenomic-based therapy with a positive outcome. Concerning the entire group, we observe that most patients responded positively to the treatment, with 5 out of 17 (29%) demonstrating partial response, 1 patient demonstrating mixed response, and 1 patient showing CR to the treatment suggested (Figure 2). On the other hand, 4 out of 17 (23,5%) patients remained stable and another 4 out of 17 developed a progressive disease (23,5%). The responses for the suggested treatments for all patients are shown in Figure 2.

### Case Study

As a case study, we report the example of a patient with Stage IV metastatic gastric adenocarcinoma with nodal and liver metastases that responded completely to the treatment (CY102, first patient in Figure 1, age range 40–50). Following the cancer diagnosis at 07/2016, the patient's samples were sent for molecular analysis at 10/2016. Based on the data obtained, this patient had a positive expression of topoisomerase 1 (TOPO1) and TOPO2A proteins. For that reason, a topoisomerase I or topoisomerase II inhibitor was highly suggested as a beneficial therapeutic regimen. Moreover, cancer cells demonstrated high expression of Programmed death-ligand 1 (PD-L1), thus denoting that a PD-1/PD-L1 inhibitor therapy would likely be associated with a positive response. Indeed, epirubicin (topoisomerase II inhibitor) was added to the therapeutic regimen in a 3-cycle EOX (epirubicin, oxaliplatin, capecitabine) chemotherapy scheme. Afterward, pembrolizumab, an anti-PD1/PD-L1 immunotherapy, was initiated with CR after the first CT re-evaluation scan. The patient had very little treatment toxicities, and thus, he started on maintenance treatment with pembrolizumab and is currently continuing maintenance therapy on a 3-weekly basis. He is currently well with a performance status of 0 and a recent PET/CT scan did not reveal any local or distance disease recurrence. By delivering this pharmacogenomics-based therapeutic strategy, the oncology team was capable of noticing that there were other treatment options that, at the time, were not recommended by any International Guidelines, but were suggested by the report, and thus, by the initiation of the suggested medication, resulted in a CR with the patient to be now in cancer-free maintenance status.

### Discussion

Providing effective and long-lasting therapeutic outcomes in clinical oncology remains a pressing medical problem worldwide. The inherent complexity of the cancer cell physiology, the tumor microenvironment, as well as the inter-individual differences are variables that highly impact therapy progression. Despite the substantial technological advances in the omics and data analysis fields, information by tumor profiling is still rarely utilized by clinicians when deciding on a potential therapy. Multiple reasons, including availability to the test, lack of information, as well cost, are still considered barriers for the implementation of the technology in the routine clinical practice (19).

Nevertheless, most studies in the field conclude that, indeed, tumor profiling represents the best approach for obtaining information that would be useful to clinicians in the routine oncology practice. While initial studies focused mainly on genomic profiling, nowadays, studies and tests include transcriptomic, proteomic, and other biomarker testing such microsatellite instability (MSI) and immunograms. Moreover, expert-guided precision oncology bases are constantly being developed, in order to connect tumor-related mutations with standardized pharmacogenomic reports (such as the OncoKB) (20) or to simulate pharmacokinetic and pharmacodynamic effects of therapeutics in the body (21). The end products of this research are commercially available tests, such as the OncoDEEP™, which offers a comprehensive combination of DNA, RNA, and protein tests, followed by data analysis and data interpretation to provide easily interpretable knowledge on a personalized manner. By that, clinicians can best decide on chemotherapy, targeted therapy, or immunotherapy, and deliver more effective, safe, and less costly therapies.

The data presented in this study further highlight the importance of molecularly guided therapeutic decisions in the clinical setting. All patient management was conducted in a private oncology center; hence, our study represents a paradigm on how molecular tumor profiling can be broadly implemented by clinicians working in small centers, worldwide. The analysis suggests that tumor profiling can improve overall disease response rates. Moreover, despite the fact that the study was conducted on a small number of patients, we observe that the earlier a pharmacogenomic-based therapy is initiated, the better and more durable the therapeutic outcome is. A limitation of this analysis is, indeed, it is relatively small scale, including only 17 patients. Nevertheless, our goal was to exactly demonstrate how tumor profiling information can provide quick and durable solutions in oncology decision-making in small- to medium-sized centers. Moreover, our case study shows how a drug that was not recommended by any International Guideline can be repurposed to target a highly aggressive type of cancer in a unique patient. This is a typical example of how personalized medicine can provide therapeutic solutions that would otherwise be neglected. In conclusion, we expect that this work will urge healthcare professionals to more broadly implement tumor profiling in their everyday clinical practice.

## Data Availability Statement

The raw data supporting the conclusions of this article will be made available by the authors, without undue reservation, to any qualified researcher.

## Ethics Statement

The studies involving human participants were reviewed and approved by The National Bioethical Committee of Cyprus. Written informed consent was obtained from all the patients included in the present study.

## Author Contributions

IV designed and coordinated the study. CP, KK, and IV performed data analysis and drafted manuscript. GA and CM conducted patient management and clinical data analysis.

## Conflict of Interest

IV declares consulting to Melidonia Health Services, Cyprus. The remaining authors declare that the research was conducted in the absence of any commercial or financial relationships that could be construed as a potential conflict of interest.
