# Supplementary material for: Pharmacogenomic Testing to Guide Personalized Cancer Medicine Decisions in Private Oncology Practice: A Case Study
Source: Front Oncol. 2020 Apr 28;10:521. doi: 10.3389/fonc.2020.00521 (PMC7199631; doi:10.3389/fonc.2020.00521)
Supplement: Supplementary file 1 [file Data_Sheet_1.PDF]

frontiers

Pharmacogenomic testing to guide personalized cancer medicine decisions in private oncology practice: A case study

George Astras[1], Christos I. Papagiannopoulos[2#], Konstantinos A. Kyritsis[2#], Constantina Markitani[1], Ioannis S. Vizirianakis[2*]

[1]American Medical Center, 215 Spyrou Kyprianou Ave., 1311 Nicosia, Cyprus

[2]Laboratory of Pharmacology, School of Pharmacy, Aristotle University of Thessaloniki, 54124 Thessaloniki, Greece

[#] Equal contribution authors

*** Correspondence:**
Ioannis S. Vizirianakis
ivizir@pharm.auth.gr

Keywords: Personal cancer genome sequencing, precision medicine, oncology, pharmacogenomic testing, targeted therapeutics, next generation sequencing, routine clinical practice

Content

1. **Supplementary Figure 1.** Heatmap displaying the number and type of mutations identified in each patient
2. **Supplementary Figure 2.** Heatmap displaying the levels of 11 known protein markers in each patient's sample
3. **Supplementary Figure 3.** Heatmap displaying the levels of 17 biomarkers in each patient's sample

Supplementary Material

1.1 Supplementary Figures

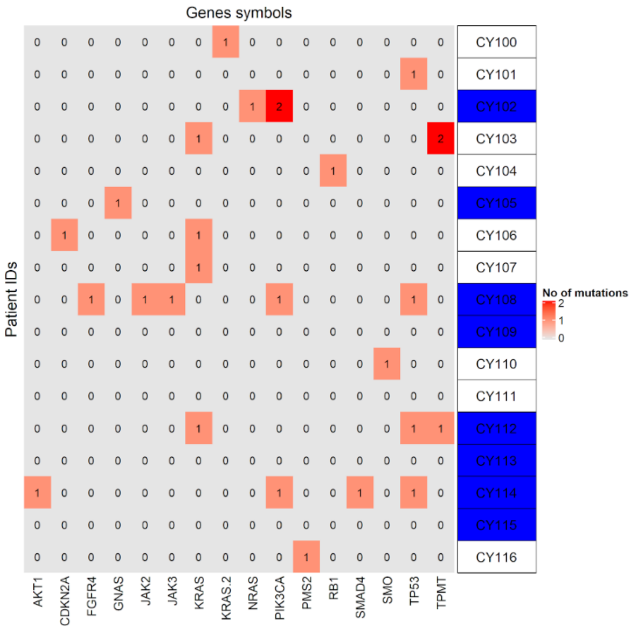

Supplementary Figure 1. Heatmap displaying the number and type of mutations identified in each patient (Horizontal axis: Gene names, Vertical axis: Patients' code). Patients that responded positively to the suggested treatment are highlighted with blue. The heatmap was created using the ComplexHeatmap R package.

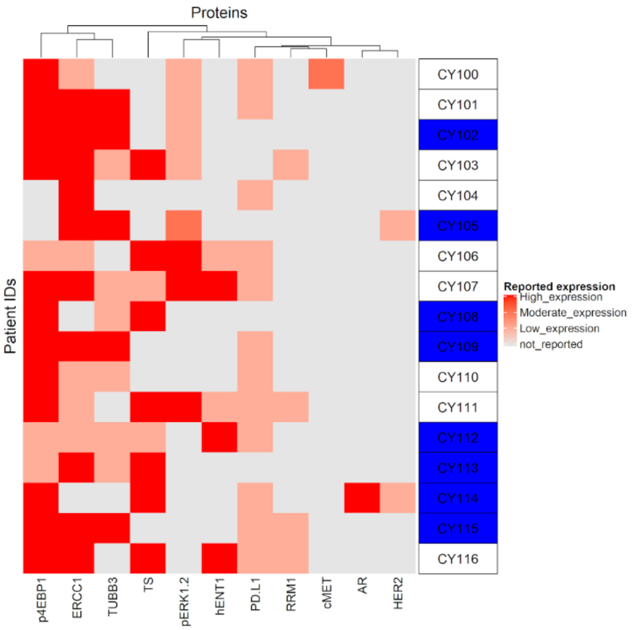

Supplementary Figure 2. Heatmap displaying the levels of 11 known protein markers in each patient's sample. Horizontal axis corresponds to protein names and vertical to patient code. Patients that showed a positive response to the suggested treatment are highlighted with blue. The heatmap was created using the ComplexHeatmap R package.

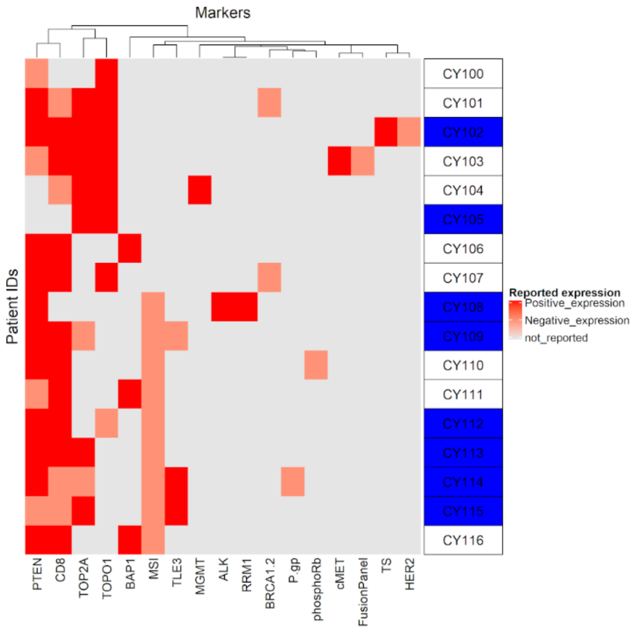

Supplementary Figure 3. Heatmap displaying the levels of 17 biomarkers in each patient's sample. Horizontal axis corresponds to biomarkers and vertical to patient code. Patients that responded favorably to the suggested treatment are highlighted with blue. Heatmap was created using the ComplexHeatmap R package
